# Impact of cell wall polysaccharide modifications on the performance of *Pichia pastoris*: novel mutants with enhanced fitness and functionality for bioproduction applications

**DOI:** 10.1186/s12934-024-02333-0

**Published:** 2024-02-17

**Authors:** Bingjie Cheng, Keyang Yu, Xing Weng, Zhaojun Liu, Xuewu Huang, Yuhong Jiang, Shuai Zhang, Shuyan Wu, Xiaoyuan Wang, Xiaoqing Hu

**Affiliations:** 1 School of Biotechnology, Jiangnan University, 1800 Lihu Road, Wuxi, Jiangsu 214122 China; 2 College of Pharmacy, Guangxi Medical University, Nanning, 530021 China; 3 State Key Laboratory of Food Science and Resources, Jiangnan University, 1800 Lihu Road, Wuxi, Jiangsu 214122 China; 4 Hopkirk Research Institute, AgResearch Ltd, Massey University, University Avenue and Library Road, Palmerston North, 4442 New Zealand

**Keywords:** *Pichia pastoris*, Cell wall, *β*-1,3-D-glucan synthase, Carbon conversion ratio, Human epidermal growth factor, *S*-adenosyl-L-methionine, Ergothioneine, ATP, Unsaturated lipids

## Abstract

**Background:**

*Pichia pastoris* is a widely utilized host for heterologous protein expression and biotransformation. Despite the numerous strategies developed to optimize the chassis host GS115, the potential impact of changes in cell wall polysaccharides on the fitness and performance of *P. pastoris* remains largely unexplored. This study aims to investigate how alterations in cell wall polysaccharides affect the fitness and function of *P. pastoris*, contributing to a better understanding of its overall capabilities.

**Results:**

Two novel mutants of GS115 chassis, H001 and H002, were established by inactivating the *PAS_chr1-3_0225* and *PAS_chr1-3_0661* genes involved in *β*-glucan biosynthesis. In comparison to GS115, both modified hosts exhibited a looser cell surface and larger cell size, accompanied by faster growth rates and higher carbon-to-biomass conversion ratios. When utilizing glucose, glycerol, and methanol as exclusive carbon sources, the carbon-to-biomass conversion rates of H001 surpassed GS115 by 10.00%, 9.23%, and 33.33%, respectively. Similarly, H002 exhibited even higher increases of 32.50%, 12.31%, and 53.33% in carbon-to-biomass conversion compared to GS115 under the same carbon sources. Both chassis displayed elevated expression levels of green fluorescent protein (GFP) and human epidermal growth factor (*hegf*). Compared to GS115/pGAPZ A-*gfp*, H002/pGAPZ A-*gfp* showed a 57.64% higher GFP expression, while H002/pPICZα A-*hegf* produced 66.76% more *hegf*. Additionally, both mutant hosts exhibited enhanced biosynthesis efficiencies of *S*-adenosyl-L-methionine and ergothioneine. H001/pGAPZ A-*sam2* synthesized 21.28% more SAM at 1.14 g/L compared to GS115/pGAPZ A-*sam2*, and H001/pGAPZ A-*egt1E* obtained 45.41% more ERG at 75.85 mg/L. The improved performance of H001 and H002 was likely attributed to increased supplies of NADPH and ATP. Specifically, H001 and H002 exhibited 5.00-fold and 1.55-fold higher ATP levels under glycerol, and 6.64- and 1.47-times higher ATP levels under methanol, respectively, compared to GS115. Comparative lipidomic analysis also indicated that the mutations generated richer unsaturated lipids on cell wall, leading to resilience to oxidative damage.

**Conclusions:**

Two novel *P. pastoris* chassis hosts with impaired *β*-1,3-D-glucan biosynthesis were developed, showcasing enhanced performances in terms of growth rate, protein expression, and catalytic capabilities. These hosts exhibit the potential to serve as attractive alternatives to *P. pastoris* GS115 for various bioproduction applications.

**Supplementary Information:**

The online version contains supplementary material available at 10.1186/s12934-024-02333-0.

## Introduction

*Pichia pastoris*, known as *Komagataella phaffii*, is a widely adopted host for industrial protein expression and metabolite production [1, 2]. Now it is extensively utilized as a host to produce various products [3], such as food and feed enzymes [4], antibodies and vaccines [5], cosmetology ingredients [6], as well as biofuels and other chemicals [7]. For examples, *P. pastoris* that was incorporated by heterologous inositol-3-phosphate synthase (IPS) and inositol monophosphates (IMP) could synthesize 30.71 g/L myo-inositol [8]. Another *P. pastoris* was employed to produce 75.48 mg/g lycopene through metabolic engineering [9]. *P. pastoris* distinguishes itself through the employment of robust promoters, such as P_AOX_ and P_GAP_, ensuring accurate gene regulation [10, 11]. The system is characterized by its user-friendly genetic manipulation capabilities and a well-established secretion system that facilitates the efficient release of proteins externally [12, 13]. Additionally, *P. pastoris* excels in post-translational modifications, and high-density fermentation, surpassing 150 g/L, which serves to significantly improve protein expression [14].

Diverse strategies have been devised to alter the performance of the *P. pastoris* host [15, 16]. Cell surface modification was undertaken by Weinhandl et al., involving the inactivation of *Cwp1*, *Scw10*, and *Och1*, resulting in heightened branched-chain aminotransferase secretion and an increase in glycosylated protein levels. The modified cell wall exhibited reduced viscosity and more permeable structures [17]. Näätsaari’s study reported that the deletion of the *KU70* homologue, a crucial DNA end-binding protein essential for NHEJ, resulted in a significant boost, achieving a remarkable increase to 90% in homologous recombination efficiency [18]. Additionally, the performance of *P. pastoris* was modified by introducing mutations to the P_GAP_ promoter [19], or through the modification of NADH oxidase (encoded by *noxE*) [20].

Given the high demand for sustainable biological manufacturing, various strategies in chassis engineering have been developed to enhance the performance of *P. pastoris* chassis cells [15]. Zhu et al. observed that overexpression of *PpSPI1*, a glycosylphosphatidylinositol-anchored cell wall glycoprotein involved in the formation of the mannoprotein layer, in Glyco4 could partially restore cell wall defects and increase resistance to cell wall disruptors and osmotic stress [21]. To date, modifications to the *P. pastoris* cell surface have been primarily focused on cell membrane proteins. However, the potential impact of alterations in cell wall polysaccharides on the fitness and function of *P. pastoris* remains unexplored.

**Fig. 1:**
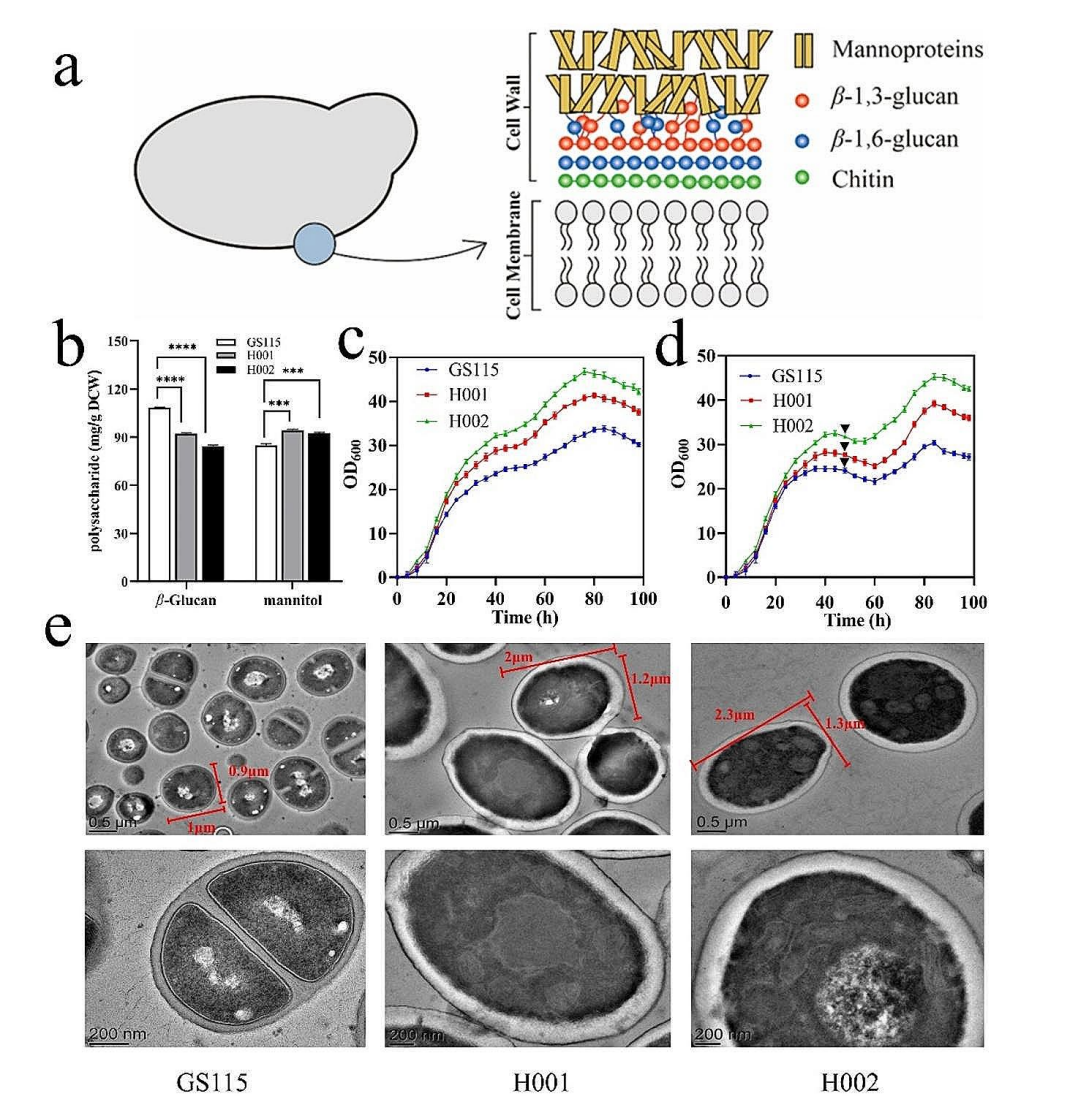
Schematic representation of cell wall structure and the effect of glucan deficiency on growth, cell wall polysaccharides contents and micromorphology. (**a**) The schematic of the structural organization of *Candida albicans* cell wall. (**b**) *β*-glucan and mannan content. (**c**) The growth curves under BMGY medium. (**d**) The growth curves under BMGY and BMMY medium. Arrows show points in time when methanol was added. (**e**) Transmission electron microscope (TEM) images. Mean ± SEM are shown (*n* = 6). Error bars indicate standard deviation. *** represented *p* < 0.001 and **** represented *p* < 0.0001

Research conducted on alternative hosts, such as *E. coli*, has indicated that surface polysaccharides can significantly influence overexpression performance. In the prior investigations, it was observed that the truncation of lipopolysaccharides on the *E. coli* cell surface resulted in enhanced host performance. This improvement was attributed to heightened carbon source utilization rates and increased outer membrane permeability [22–24]. While the cell wall structure of *P. pastoris* has not been extensively studied, it is recognized that in other yeasts, such as *Saccharomyces cerevisiae* and *Candida albicans* (Fig. 1a), the predominant polysaccharides—*β*-glucans and mannans—comprise 85-90% of the cell wall’s dry weight [25–27]. Inactivation of *β*-1,3-D-glucan synthase in *S. cerevisiae* has been shown to reduce glucan content by approximately 55%, although its impact on host performance remains unstudied [28, 29].

It is hypothesized that *P. pastoris* GS115 produces excess cell wall polysaccharides, resulting in disproportionate carbon source utilization. Hypothetically, reducing these polysaccharides could improve carbon efficiency and utilize resources for growth, protein expression, and biocatalysis. Therefore, this study is to investigate the potential impact of modifying *β*-glucans and mannans within the cell wall on the *P. pastoris* chassis host.

## Material and method

### Strains, plasmids, primers and strain cultivation

The strains, plasmids, and primers used are listed in Table 1 and Additional files 5–6 (Table S1, Table S2). *E. coli* JM109, *P. pastoris* GS115, plasmid pGAPZα A, pGAPZ A, pPICZα A and pPICZ A, were purchased from Invitrogen (California, USA). The plasmid pPpT4_pHTX1-hsCas9 was synthesized based on the reported sequences [30].

**Table 1:** The host and recombinant strains used in the present study

Strains	Descriptions
**Host strains**	
*Escherichia coli* JM109	wild-type *E. coli*
*Pichia pastoris* GS115	wild-type *Pichia pastoris*
H001	GS115 mutant in *PAS_chr1-3_0225* gene
H002	GS115 mutant in *PAS_chr2-1_0661* gene
**GFP-expressing strains**	
GS115*-*pGAPZ A*-gfp*	GS115 harboring *gfp* under P_GAP_
GS115*-*pPICZ A*-gfp*	GS115 harboring *gfp* under P_AOX_
H001*-*pGAPZ A*-gfp*	H001 harboring *gfp* under P_GAP_
H002*-*pGAPZ A-*gfp*	H002 harboring *gfp* under P_GAP_
H001*-*pPICZ A*-gfp*	H001 harboring *gfp* under P_AOX_
H002*-*pPICZ A-*gfp*	H002 harboring *gfp* under P_AOX_
***hegf*** **-expressing strains**	
GS115*-*pGAPZα A*-hegf*	GS115 harboring *hegf* under P_GAP_ and α factor
GS115*-*pPICZα A*-hegf*	GS115 harboring *hegf* under P_AOX_ and α factor
H001*-*pGAPZα A*-hegf*	H001 harboring *hegf* under P_GAP_ and α factor
H002*-*pGAPZα A-*hegf*	H002 harboring *hegf* under P_GAP_ and α factor
H001*-*pPICZα A*-hegf*	H001 harboring *hegf* under P_AOX_ and α factor
H002*-*pPICZα A-*hegf*	H002 harboring *hegf* under P_AOX_ and α factor
**SAM-biosynthesis strains**	
GS115*-*pGAPZ A*-sam2*	GS115 harboring *sam2* under P_GAP_
GS115*-*pPICZ A*-sam2*	GS115 harboring *sam2* under P_AOX_
H001*-*pGAPZ A*-sam2*	H001 harboring *sam2* under P_GAP_
H002*-*pGAPZ A-*sam2*	H002 harboring *sam2* under P_GAP_
H001*-*pPICZ A*-sam2*	H001 harboring *sam2* under P_AOX_
H002*-*pPICZ A-*sam2*	H002 harboring *sam2* under P_AOX_
**ERG-biosynthesis strains**	
GS115*-*pGAPZ A*-egt12*	GS115 harboring *egt12* under P_GAP_
GS115*-*pGAPZ A*-egt1E*	GS115 harboring *egt1E* under P_GAP_
GS115*-*pPICZ A*-egt12*	GS115 harboring *egt12* under P_AOX_
GS115*-*pPICZ A*-egt1E*	GS115 harboring *egt1E* under P_AOX_
H001*-*pGAPZ A*-egt12*	H001 harboring *egt12* under P_GAP_
H001*-*pGAPZ A*-egt1E*	H001 harboring *egt1E* under P_GAP_
H001*-*pPICZ A*-egt12*	H001 harboring *egt12* under P_AOX_
H001*-*pPICZ A*-egt1E*	H001 harboring *egt1E* under P_AOX_
H002*-*pGAPZ A*-egt12*	H002 harboring *egt12* under P_GAP_
H002*-*pGAPZ A*-egt1E*	H002 harboring *egt1E* under P_GAP_
H002*-*pPICZ A*-egt12*	H002 harboring *egt12* under P_AOX_
H002*-*pPICZ A*-egt1E*	H002 harboring *egt1E* under P_AOX_

*E. coli* was incubated in Luria-Bertani (LB) medium (5 g/L yeast extract, 10 g/L tryptone, and 10 g/L NaCl) at 37 ℃.

YPD medium for inoculum culture of *P. pastoris* contained: 1 g/L yeast extract, 2 g/L peptone, and 2 g/L glucose. For production, P_GAP_-driven *P. pastoris* was cultured in 50 mL BMGY medium [10 g/L glycerol, 10 g /L yeast extract, 20 g/L peptone, 13.4 g/L YNB, 100 mM K_3_PO_4_ buffer, 5 g/L (NH_4_)_2_SO_4_ and 400 µg/L biotin, pH 6.0] at 30 ℃ and 220 rpm. After the exhaustion of glycerol, 1 mL of 50% glycerol solution was added every 24 h. P_AOX_-driven *P. pastoris* was initially cultured in 50 mL of BMGY medium for 2 d, and later transferred into 50 mL of BMMY medium [10 g/L methanol, 10 g/L yeast extract, 13.4 g/L YNB, 20 g/L peptone, 5 g /L (NH_4_)_2_SO_4_, biotin 400 µg, 100 mmol K_3_PO_4_ buffer, pH 6.0]. In the induction phase, 1% methanol was added every 24 h. All the culture of *P. pastoris* was conducted at 30 ℃ and 220 rpm.

### Construction of glucan-deficient chassis hosts

According to the reported procedure [31], *PAS_chr1-3_0225* and *PAS_chr1-3_0661* genes involved in glucan synthesis were knocked out as follows. Firstly, 100 ng of plasmid pPpT4_pHTX1-hsCas9 and 1 µg of donor DNA fragments were co-transformed into competent cells of GS115. All corresponding sgRNA and primers were designed based on the genome sequence of *P. pastoris* GS115(Additional file 6: Table S2). Later, transformants were selected on YPD agar plates containing 100 mg/L Zeocin, and the genomic DNA was isolated following the protocol [32]. Finally, the accuracy of gene inactivation was confirmed through PCR, DNA electrophoresis, and gene sequencing.

### Plasmid construction

Using the ClonExpress II One Step Cloning Kit (Vazyme, Suzhou, China), *gfp* gene was inserted downstream of P_GAP_ in pGAPZ A, generating pGAPZ A-*gfp*. Similarly, *gfp* was also inserted downstream of P_AOX_, resulting in pPICZ A-*gfp* (Additional file 1: Fig. S1). Subsequently, both pGAPZ A-*gfp* and pPICZ A-*gfp* were transformed into *E. coli* JM109, respectively. The correct transformations were confirmed through PCR, DNA electrophoresis and gene sequencing.

The *hegf* gene (Gene ID: NP_001171601.1) was codon-optimized and synthesized by GENEWIZ (Suzhou, China) to facilitate its expression in *P. pastoris*. 6 × His tag was added at the 3’ end of the sequence. The expression plasmids pGAPZα A and pPICZα A were employed, and the *hegf* gene was then inserted downstream of the α-factor signal peptide to construct pGAPZα A-*hegf* and pPICZα A-*hegf* (Additional file 1: Fig. S1) respectively, similar to the procedure mentioned above for GFP.

Similarly, *sam2* gene, encoding SAM synthetase (Gene ID: 852,113), was amplified from *S. cerevisiae* S288C and inserted into pGAP A and pPICZ A (Additional file 1: Fig. S1).

To biosynthesize ERG, two biosynthesis pathways employing different genes (*egt1*2 and *egt1E*) were designed as follows. Firstly, *egt1* (Gene ID: 3,872,471) and *egt2* (Gene ID: 2,828,225) from *Verticillium rougheri*, and *egtE* (Gene ID: 66,737,529) from *Mycobacterium pubescens* were codon-optimized to facilitate expression in *P. pastoris* and synthesized by GENEWIZ (Suzhou, China). Later, pGAP A and pPICZ A were used to construct 4 recombinant plasmids: pGAPZ A-*egt1*2, pGAPZ A-*egt1E*, pPICZ A-*egt1*2, and pPICZ A-*egt1E* (Additional file 1: Fig. S1).

### Construction of recombinant strains

The plasmids carrying P_GAP_ were digested by AvrII fast digestion enzyme, while those carrying P_AOX_ were digested using SacI fast digestion enzyme. Later, the plasmids were purified using a DNA product purification kit (Vazyme, Suzhou, China) to obtain linearized plasmids, which were electroporated into GS115, GS115Δ*PAS_chr1-3_0225* (named as H001), and GS115Δ*PAS_chr2-1_0661* (named as H002) respectively. Following electroporation, single colonies were chosen from YPD plates supplemented with 300 µg/mL bleomycin. Gene sequencing was conducted to verify the correct transformants.

### Cell growth examination

The optical density at 600 nm (OD_600_) and wet cell weight (WCW) were measured according to the previous methods [33]. Glycerol was assayed by the reported method [34]. The ratios of glucose, glycerol, and methanol converted to biomass were calculated based on the consumption of glucose, glycerol, methanol, and WCW, respectively. The residual glucose level was determined by Automatic Residual Sugar Analyzer (SBA-40 C, Shandong Academy of Sciences, China).

### The detection of mannan and *β*-glucan

To detect mannan and *β*-glucan, 5 g of yeast cells was harvested, then subjected to 100 mL of SDS-LiAc lysate (50 mmoL/L LiAc, 0.034 mol/L SDS) and incubated at 70 °C for 10 min. The resulting mixture was centrifuged and the suspension was mixed with 5 mL of 95% ethanol to precipitate the polysaccharides. The resulting sediment was freeze-dried and divided into two parts for further extraction. One part was treated with 72% (v/v) H_2_SO_4_, while the other part was treated with a solution containing 3% (w/v) NaOH and 6% (v/v) glacial acetic acid. Both parts were finally washed with ethanol and the resultant precipitate was detected by high performance liquid chromatography (HPLC) reported previously [35].

### Cell morphology examination

To observe the micromorphology change, *P. pastoris* cells in mid-exponential phase were harvested by centrifugation, and then suspended in a 2% glutaraldehyde fixative after being washed three times with a 0.1 M phosphate buffered saline (PBS). The treated cells were observed using TEM (Jem2100, Japan Electronics) [36].

### Fluorescence detection of GFP expression

GFP expression was detected by the previous fluorescence microscope method [37], with minor modification as follows. Briefly, the cells in the logarithmic phase were collected and placed on a slide. Then a sealer (anti-fluorescence attenuation) was added, and GFP fluorescence was observed under the autofluorescence microscope (TCS SP8, Leica, Germany). To measure fluorescence intensity, fermentation of mid-logarithmic cells was taken, then washed three times with PBS, and later suspended in PBS to obtain OD_600_ at 0.2. Subsequently, 200 µL of cell suspension was transferred to a 96-well plate. The fluorescence intensity was read at excitation wavelength of 488 nm and emission wavelength of 525 nm using Cytoation5 ELISA GFP measurement program. The fluorescence value of *P. pastoris* cell not carrying *gfp* was used as control.

### Extraction of expressed proteins

The extraction and detection methods for *hegf* employ in this study were identical to that reported previously [38, 39]. His-tagged *hegf* protein was extracted from culture supernatant using a protein purification kit (Sangon Biotech, Shanghai). The protein electrophoresis procedure was identical to the previous publication [40]. SAM was extracted and measured using our previously established method [41, 42].

### Detection of cell metabolism

ATP was assayed by a procedure described previously [43]. NADPH was detected using the NADP^+^/NADPH Assay Kit with WST-8 (Beyotime, Shanghai, China). ERG was extracted and determined using the reported procedure [44]. The content of malondialdehyde (MDA), a lipid peroxidation product, was determined using the MDA assay kit (Sangon Biotech, Shanghai, China) [45]. The global lipid extraction and lipidomics analysis were conducted following the references [46, 47].

## Results

### Phenotypic changes of new chassis cells

Using BLAST in NCBI, *PAS_chr1-3_0225* from *P. pastoris* GS115 showed 98% protein identity with FKS2 from *S. cerevisiae*, while *PAS_chr1-3_0661* showed 97% protein identity with FKS1 from *S. cerevisiae* [28, 48]. Both genes likely encoded *β*-1,3-D-glucan synthases and were thus inactivated respectively, creating two mutants: GS115-Δ*PAS_chr1-3_0225* (H001) and GS115-Δ*PAS_chr2-1_0661* (H002).

To confirm the phenotypic change, glucan content was assayed. In the logarithmic growth phase, GS115 showed 108.32 mg glucan/g WCW, while H001 and H002 exhibited 17.45% and 28.33% lower levels at 92.23 and 84.41 mg/g respectively. The results indicated that the knockout of *PAS_chr1-3_0225* or *PAS_chr1-3_0661* partially inhibited glucan biosynthesis as anticipated. Furthermore, change in mannan, another major polysaccharide within *P. pastoris* cell wall, was also quantified. H001 and H002 showed 94.24 and 92.52 mg mannan/g WCW, 10.94% and 8.91% higher than GS115 (84.95 mg/g), respectively (Fig. 1b).

Later, the microscopic changes of 2 chassis hosts were observed through TEM. Compared to GS115, H001 and H002 displayed thicker but brighter cell surfaces, indicating looser structure of cell walls. The size of the representative cells of 3 hosts were marked respectively (Fig. 1e). The cell of GS115 was in length 1.0 μm with a diameter of 0.9 μm, however, H001 showed a larger cell (length 2.0 μm with a diameter of 1.2 μm), and H002 displayed the largest size (length 2.3 μm and diameter 1.3 μm).

Rapid growth rate was essential for chassis host, thus the growth curves of GS115, H001 and H002 under BMGY and BMMY media were compared. For either promoter, both H001 and H002 displayed higher growth rates and achieved higher peak biomass compared to GS115 (Fig. 1c-d). Under BMGY media (Fig. 1c), H001 and H002 achieved the maximal OD_600_ of 41.35 at 80 h and 46.78 at 76 h respectively, 22.41% and 38.48% higher than the corresponding value of GS115 (OD_600_ of 33.78 at 84 h). Similar improvements were observed for P_AOX_-*Pichia* under medium BMGY + BMMY (Fig. 1d). In the batch phase under BMGY, both H001 and H002 consumed 2% glycerol after 44 h, obtaining OD_600_ at 29.31 and 32.67 respectively, 19.29% and 32.97% greater than GS115 (OD_600_ of 24.57 at 44 h). During the subsequent induction phase under BMMY, H001 achieved the peak OD_600_ of 39.21 after 84 h, 29.11% higher than that of GS115 (OD_600_ at 30.37), while H002 achieved the highest value at 45.27 after 84 h, 49.06% higher than GS115 (Fig. 1d).

Finally, the conversion ratio of carbon sources (glucose, glycerol and methanol) to biomass, a crucial parameter for industrial biotechnology, was evaluated under YPD, BMGY and BMMY media respectively. The culture broth was collected at 12-h intervals to measure the residual glucose, glycerol and WCW. Under YPD medium, both modified hosts showed significantly higher glucose-to-biomass yields than GS115. H001 and H002 achieved their highest ratios at 0.44 g/g and 0.53 g/g after 48 h respectively, 10.00% and 32.50% higher than that of GS115 (0.40 g/g) (Table 2). The similar improvement was also achieved for BMGY medium, under which H001 and H002 reached the peak values of 0.71 g/g at 36 h and 0.73 g/g at 24 h, significantly higher than GS115 (0.65 g/g). In BMMY medium, H001 and H002 reached peak values of 0.20 g/g at 84 h and 0.23 g/g after 84 h, significantly higher than GS115 (0.15 g/g). One prominent advantage of H001 and H002 was the notable increase in the conversion ratio of carbon sources, a significant cost factor in yeast fermentation industry. Especially, H002 exhibited the most significant improvement.

**Table 2:** The conversion ratio of glycerol-to-biomass of *P. pastoris* GS115, H001 and H002

Host	Conversion rate of glycerol to biomass (g/g)			
	12 h	24 h	36 h	48 h
YPD media				
GS115	0.28 ± 0.01	0.41 ± 0.01	0.41 ± 0.01	0.40 ± 0.01
H001	0.33 ± 0.01	0.42 ± 0.01	0.43 ± 0.01	0.44 ± 0.01
H002	0.34 ± 0.00	0.48 ± 0.01	0.51 ± 0.01	0.53 ± 0.02
BMGY media				
GS115	0.38 ± 0.00	0.55 ± 0.01	0.65 ± 0.01	0.57 ± 0.01
H001	0.40 ± 0.01	0.62 ± 0.01	0.71 ± 0.01	0.59 ± 0.01
H002	0.62 ± 0.01	0.73 ± 0.01	0.73 ± 0.02	0.65 ± 0.01

### Performance evaluation of new chassis hosts

#### GFP expression

**Fig. 2:**
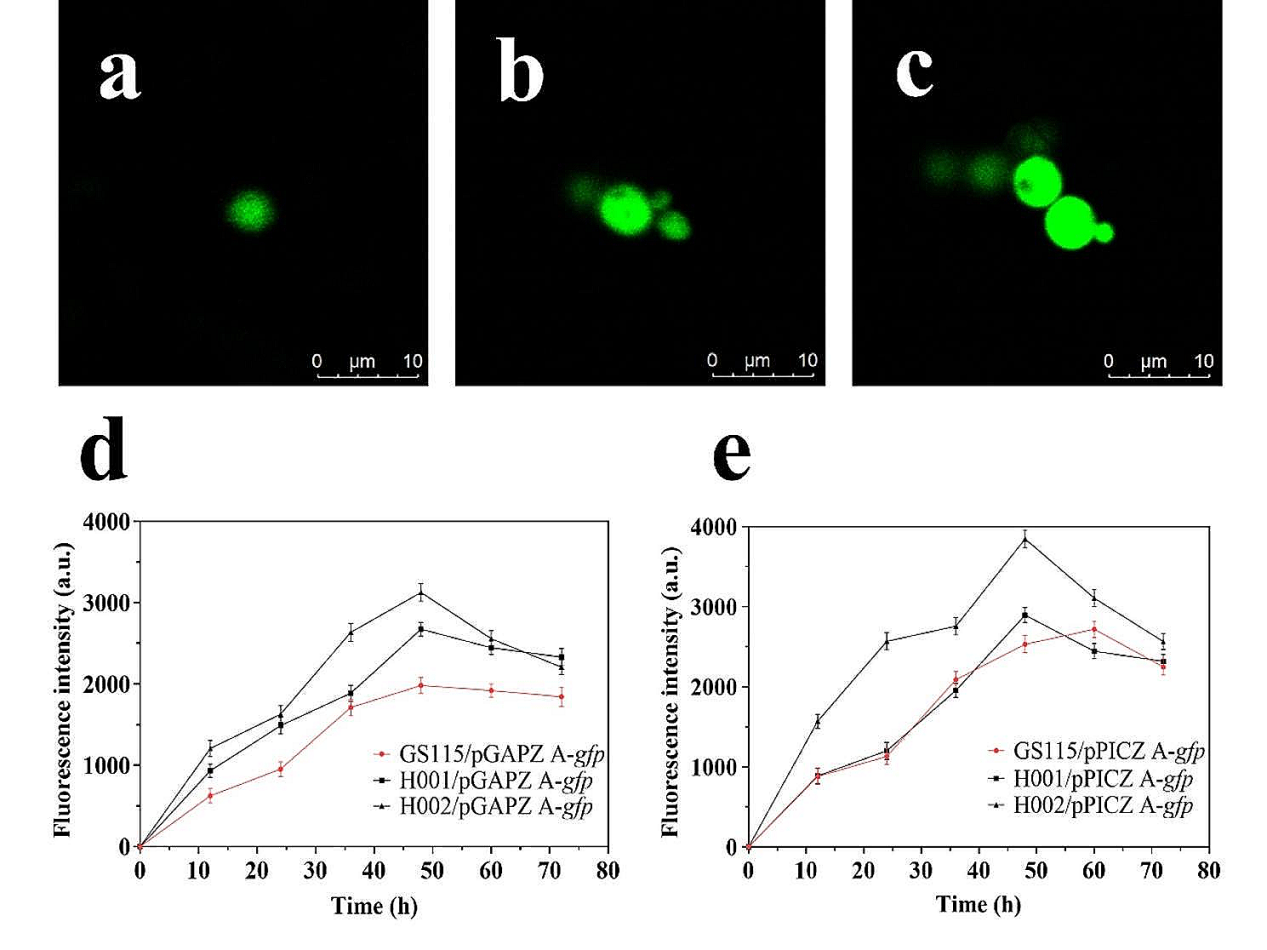
The GFP fluorescence (**a-c**) and intensity (**d-e**) of *P. pastoris* GS115, H001 and H002. (**a**) GS115/pGAPZ A-*gfp*. (**b**) H001/pGAPZ A-*gfp*. (**c**) H002/pGAPZ A-*gfp*. (**d**) P_GAP_-*Pichia*. (**e**) P_AOX_-*Pichia*. Error bars indicate standard deviation

GFP, a reporter protein employed widely, was employed at first to evaluate the chassis host performance. The fluorescence microscope revealed noticeable differences in GFP expression level between GS115 and the two glucan-reduced hosts, and the fluorescence brightness followed the order of H002 > H001 > GS115 (Fig. 2abc), which was also supported by fluorescence intensity measurements. For constitutive expression manner, H001/pGAPZ A-*gfp* exhibited 34.90% higher intensity, and H002/pGAPZ A-*gfp* showed 57.64% greater expression than GS115 (Fig. 2d). A similar trend was observed for the inducible expression manner, H001/pPICZ A-*gfp* and H002/pPICZ A-*gfp* exhibited 6.48% and 41.50% higher intensity than GS115, respectively (Fig. 2e). Overall, the reduction of glucan in H001 and H002 stimulated GFP expression, regardless of whether constitutive or inducible promoter were used.

#### *Hegf* expression

**Fig. 3:**
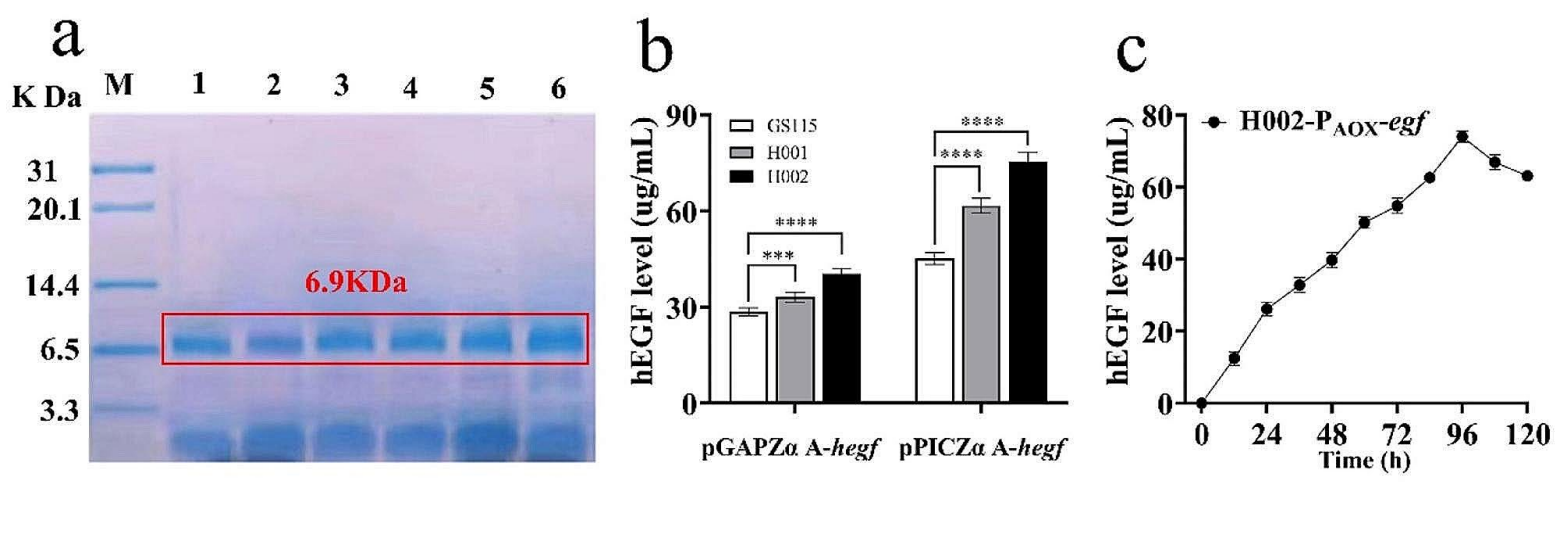
Expression of *hegf* in *P. pastoris* GS115, H001 and H002 under P_AOX_ and P_GAP_ respectively. (**a**) SDS-PAGE analysis of purified His-tagged *hegf*. lane M: protein marker; lane 1: GS115/pGAPZα A-*hegf*; lane 2: H001/pGAPZα A-*hegf*; lane 3: H002/pGAPZα A-*hegf*; lane 4: GS115/pPICZα A-*hegf*; lane 5: H001/pPICZα A-*hegf*; lane 6: H002/pPICZα A-*hegf*. (**b**) The highest *hegf* contents quantified by high-performance chromatographic liquid chromatography. (**c**) Changes in *hegf* content secreted by H002-pPICZ α A-*hegf* during cultivation. Mean ± SEM are shown (*n* = 6). Error bars indicate standard deviation. *** represented *p* < 0.001 and **** represented *p* < 0.0001

The expression of another protein *hegf* was also assessed, and 6 recombinant strains were constructed for *hegf* expression, including GS115, H001 and H002 transformed with constitutive pGAPZα A-*hegf* and inducible pPICZα A-*hegf* respectively. All strains produced the predominant 6.9-KDa protein, consistent with the expected size of the his-tagged *hegf* (Fig. 3a). Among 3 hosts, regardless either promoter (P_GAP_ or P_AOX_), H001 exhibited higher *hegf* expression than GS115, and H002 achieved the highest expression level.

Later, *hegf* was quantified by HPLC, and the results supported that from Tris-Tricine-SDS-PAGE (Fig. 3b). In P_GAP_-driven *Pichia*, H002/pGAPZα A-*hegf* produced the highest *hegf* at 40.37 µg/mL, followed by H001/pGAPZα A-*hegf* expressing 33.11 µg/mL. Their levels were 41.40% and 15.97% higher than GS115 (28.55 µg/mL), respectively. Similarly, in P_AOX_-based *Pichia* (Fig. 3c), H002/pPICZα A-*hegf* and H001/pPICZα A-*hegf* produced 66.76% and 36.67% higher *hegf* than GS115, respectively.

#### SAM biosynthesis

**Fig. 4:**
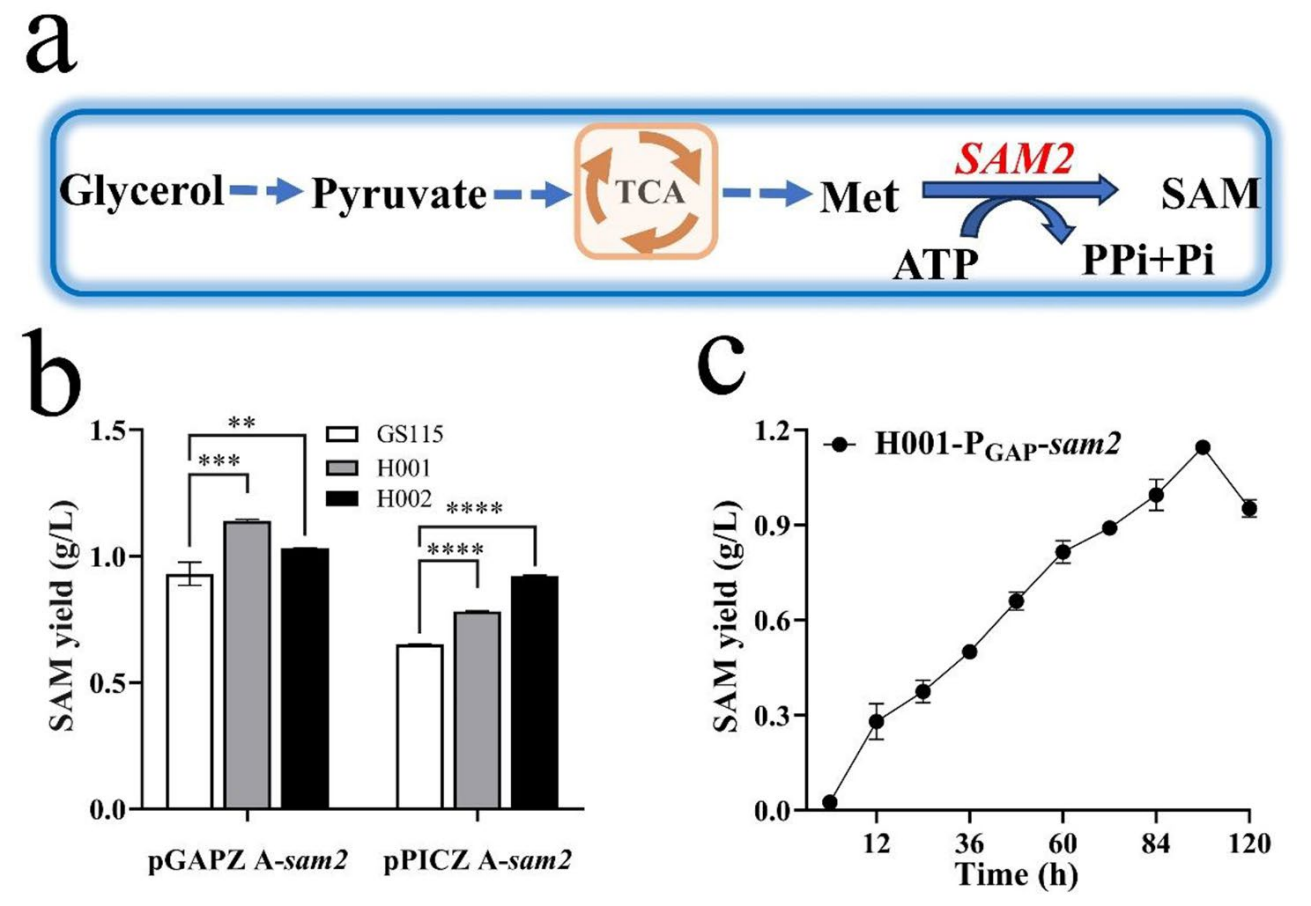
Biosynthesis level of SAM in *P. pastoris* GS115, H001 and H002 under P_AOX_ and P_GAP_ respectively. (**a**) Schematic representation of SAM biosynthesis, (**b**) The maximum SAM level. (**c**) Changes of SAM level by H001/pGAPZ A-*sam2* during cultivation. Mean ± SEM are shown (*n* = 6). Error bars indicate standard deviation. ** represented *p* < 0.01, *** represented *p* < 0.001 and **** represented *p* < 0.0001

SAM can be efficiently synthesized by *P. pastoris* overexpressing SAM synthetase (Fig. 4a). To assess the performance of the two host mutants, the biosynthesis of SAM was investigated in the following analysis.

Six strains, including GS115, H001 and H002 transformed with pGAPZ A-*sam2* and pPICZ A-*sam2* respectively, were cultured in shaking flasks using BMGY and BMMY media to induce SAM synthetase expression and initiate SAM synthesis. In the constitutive strains, H001/pGAPZ A-*sam2* produced 1.14 g/L SAM, followed by 1.03 g/L SAM by H002/pGAPZ A-*sam2* (Fig. 4c). Their yields were 21.28% and 9.57% higher than GS115/pGAPZ A-*sam2*. In the inducible strains, H002/pPICZ A-*sam2* and H001/pPICZ A-*sam2* produced 0.92 g/L and 0.78 g/L SAM respectively (Fig. 4b), 41.54% and 20.00% higher than GS115/pPICZ A-*sam2* (0.65 g/L).

**Fig. 5:**
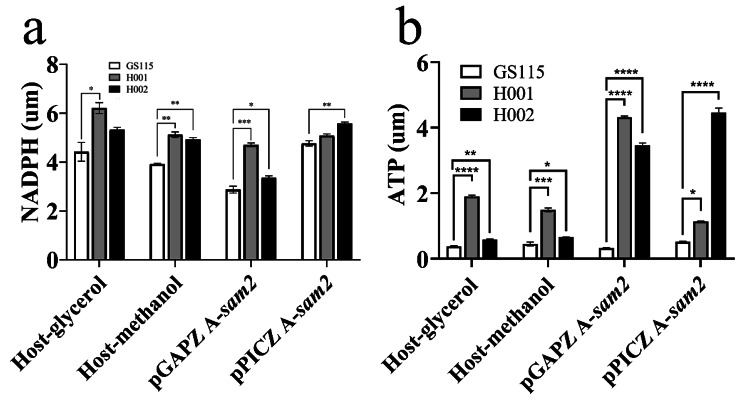
ATP and NADPH determination results of host stains (GS115、H001、H002) and SAM synthetic strains (GS115/H001/H002/pGAPZ A-*sam2*, GS115/H001/H002/pPICZ A-*sam2*). (**a**) NADPH determination result. Mean ± SEM are shown (*n* = 6). Error bars indicate standard deviation. * Represented *p* < 0.05, ** represented *p* < 0.01, *** represented *p* < 0.001 and **** represented *p* < 0.0001. (**b**) ATP determination result

In the previous reports [42, 49], ATP was identified as a limiting factor in SAM biosynthesis, therefore the intracellular ATP level, as well as the energy status, were measured. H001/pGAPZ A-*sam2* and H002/pGAPZ A-*sam2* generated 4.32 and 3.46 μM ATP respectively, 13.09-fold and 10.48-fold greater than that of GS115/pGAPZ A-*sam2* (0.33μM). In the case of H001/pPICZ A-*sam2* and H002/pPICZ A-*sam2*, their ATP levels were 1.14 and 4.46 μM, respectively, which were 2.19 and 8.58 times higher than GS115/pPICZ A-*sam2* (0.52 μM) (Fig. 5b).

#### ERG biosynthesis

**Fig. 6:**
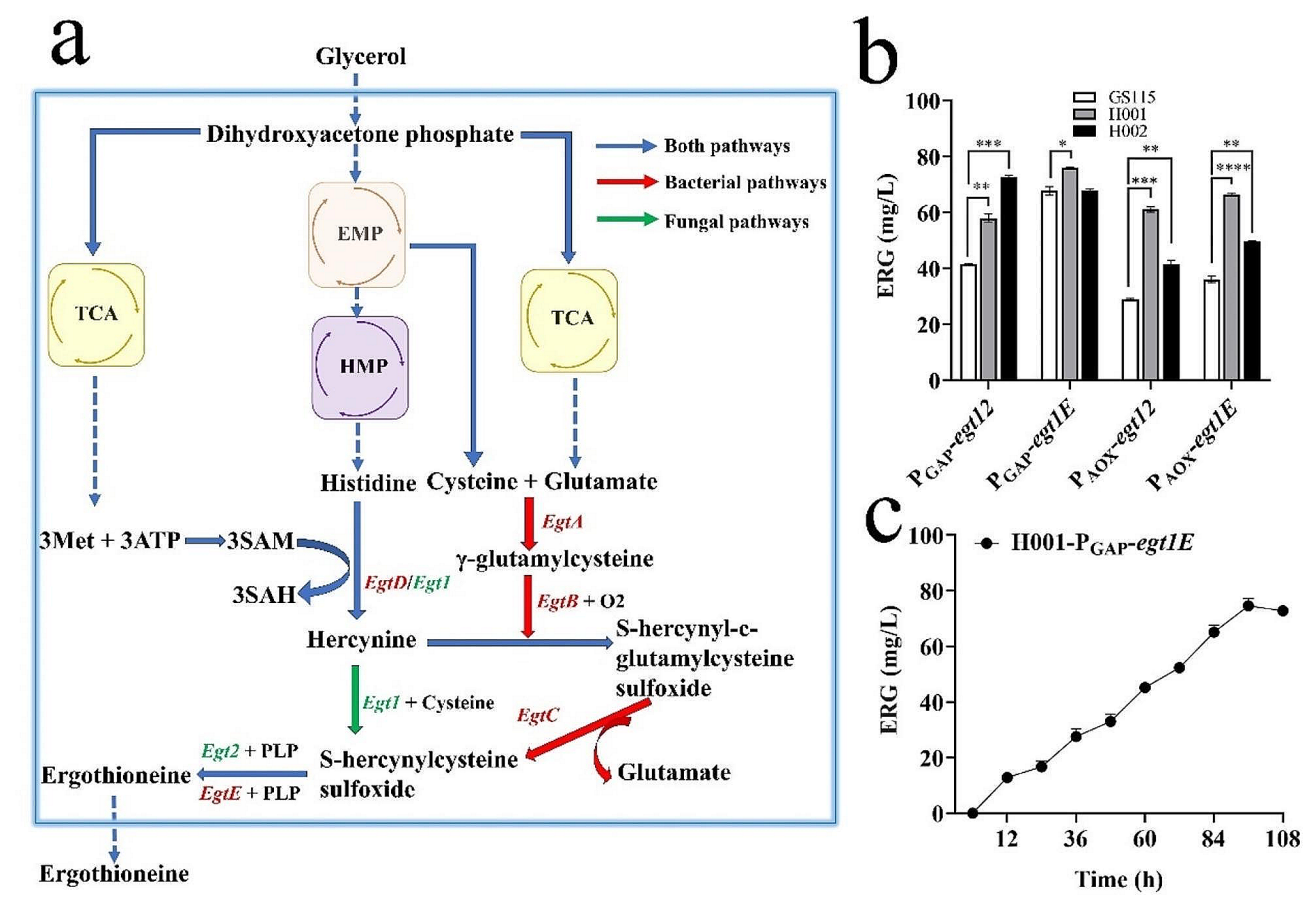
Expression of ergothioneine (ERG) in *P. pastoris* GS115, H001 and H002 under P_AOX_ and P_GAP_ respectively. (**a**) Two different pathways for ERG biosynthesis in bacteria and fungi respectively. *EgtABCDE* are responsible for ERG synthesis in bacteria, and *Egt12* are responsible for ERG synthesis in fungi. Histidine (His); Hercynine (HER); Glutamate (Glu); Cysteine (Cys); γ-glutamylcysteine (γGC); S-hercynyl-c-glutamylcysteine sulfoxide (γGC-HER); S-hercynylcysteine sulfoxide (Cys-HER); Ergothioneine (ERG). (**b**) The highest ERG levels of recombinant strains employing different chassis hosts and gene combinations. (**c**) Changes in ERG of H001-pGAPZ A-P_GAP_*-egt1E* during cultivation. Mean ± SEM are shown (*n* = 6). Error bars indicate standard deviation. * Represented *p* < 0.05, ** represented *p* < 0.01, *** represented *p* < 0.001 and **** represented *p* < 0.0001

The different gene combinations for ERG synthesis were investigated to further evaluate the performance of mutants H001 and H002 as biocatalyst expression system, (Fig. 6a). Two ERG synthesis pathways were constructed by introducing *egt1*2 and *egt1E*, respectively. There were 12 recombinant strains used, consisting of GS115, H001, and H002 transformed with pGAPZ A-*egt1*2, pGAPZ A-*egt1E*, pPICZ A-*egt1*2 and pPICZ A-*egt1E* respectively. The results showed that both novel hosts, regardless of promoter type and gene combination, were superior to GS115. When *egt1*2 was controlled by P_GAP_, H001 and H002 produced 31.11% and 43.05% higher ERG than GS115, respectively. Furthermore, when driven by P_AOX_, H002 achieved the highest ERG yield for both *egt1*2 and *egt1E*, while H001 exhibited the second level. Similarly, when *egt1E* was controlled by P_GAP_, H001 and H002 synthesized 75.85 mg/L and 67.69 mg/L ERG, 45.41% and 38.82% higher than GS115 respectively (Fig. 6b-c).

Overall, H001 and H002 performed better than GS115 for either GFP and *hegf* expression or SAM and ERG synthesis. It was still unclear why H001 and H002 deficient in glucan synthesis stimulated growth rate, protein and metabolites levels. To elucidate the underlying mechanisms, the possible factors, such as cofactors (NADPH), ATP and lipids, were further analysed in the following section.

### The higher intracellular levels of NADPH and ATP

NADPH participated in various biosynthesis processes as a common cofactor and played a critical role in the cellular response to oxidative stress. In general, the NADPH content in both modified hosts was higher than in GS115, irrespective of the promoter type (Fig. 5a). For examples, under glycerol as sole carbon source, H001 and H002 generated 6.22 μM and 5.33 μM NADPH levels respectively, 1.41-fold and 1.21-fold higher than that of GS115 (4.42 μM). Similarly, under methanol as carbon source, H001 and H002 produced NADPH at 5.12 μM and 4.92 μM respectively, 1.31 and 1.26 times richer than that of GS115 (3.92 μM). For SAM-synthesizing strains, similar enhancement was observed for the two novel hosts. H001/pGAPZ A-*sam2* and H002/pGAPZ A-*sam2* generated 1.64-fold and 1.17-fold higher NADPH levels than GS115/pGAPZ A-*sam2*, and H001/pPICZ A-*sam2* and H002/pPICZ A-*sam2* generated 1.07- and 1.17-times higher NADPH levels than GS115/pPICZ A-*sam2* respectively.

ATP was another crucial factor influencing protein expression and biocatalysis. Therefore, the intracellular ATP content was also measured. H001 and H002 utilizing glycerol as a carbon source produced 1.90 μM and 0.59 μM ATP respectively, 5.00 and 1.55 times higher than that of GS115 (0.38 μM). Similar improvement was also achieved for H001 and H002 using methanol as carbon source. For SAM-producing strains, H001/pGAPZ A-*sam2* and H002/pGAPZ A-*sam2* generated 13.09- and 10.48-times higher ATP levels than GS115/pGAPZ A-*sam2*, and H001/pPICZ A-*sam2* and H002/pPICZ A-*sam2* produced 2.19- and 8.58-times higher ATP than GS115/pPICZ A-*sam2* (Fig. 5b).

### Attenuation of oxidative damage and increase of unsaturated lipids

The oxidative stress could significantly impact the physiological state and cellular performance of *P. pastoris*. MDA is generated upon oxidative stress and was commonly used as an indicator of cellular oxidative stress [50]. The intracellular MDA level rose gradually along with cell growth, reaching the peak level in the late stationary phase for P_GAP_-*Pichia* and in the methanol induction phase for P_AOX_-*Pichia*, indicating continuous oxidative stress during the cultivation process (Additional file 2: Fig. S2). Interestingly, both H001 and H002 showed lower MDA levels than GS115, with significant differences observed when using either glycerol or methanol as the carbon source. For examples, for P_GAP_-*Pichia*, MDA levels of H001 and H002 at 64 h (the late stationary phase) were 11.50% and 16.52% less than GS115, respectively (Fig. S2a). For P_AOX_-*Pichia*, MDA levels of H001 and H002 at 64 h during the induction phase were 26.89% and 34.24% lower than GS115 (0.79 µmol/g), respectively (Additional file 2: Fig. S2b). These differences indicated lower cellular oxidative damage in H001 and H002.

Exploring the mechanism behind lower oxidative damage in glucan-deficient hosts involves considering the potential reduction in oxidative stress due to higher NADPH levels. Additionally, the close relationship between cellular oxidative damage and lipid profiles prompted a comparative lipidomic analysis of GS115, H001, and H002 to confirm this hypothesis.

Surprisingly, significant variations in lipid composition and relative intensity were observed between GS115 and the two modified hosts (Additional file 3: Fig. S3 and Additional file 7: Table S3). A total of 991 lipids were discovered, which were categorized into 3 lipid groups: (1) glycerophospholipids, including phosphatidic acid (PA), phosphatidylcholine (PC), phosphatidylethanolamine (PE), phosphatidylglycerol (PG), phosphatidylinositol (PI) and phosphatidylserine (PS); (2) glycerolipids, including diglycerides (DG) and triglycerides (TG); and (3) sphingolipids, including ceramide (Cer) and sphingomyelin (So). There were notable alterations in lipid composition in both chassis hosts (Additional file 4: Fig. S4). For example, in comparison to GS115, H001 exhibited a 30.26% increase in PCs, and H002 showed a 22.21% increase, along with 2.09% and 0.88% increases in PSs, respectively.

**Fig. 7:**
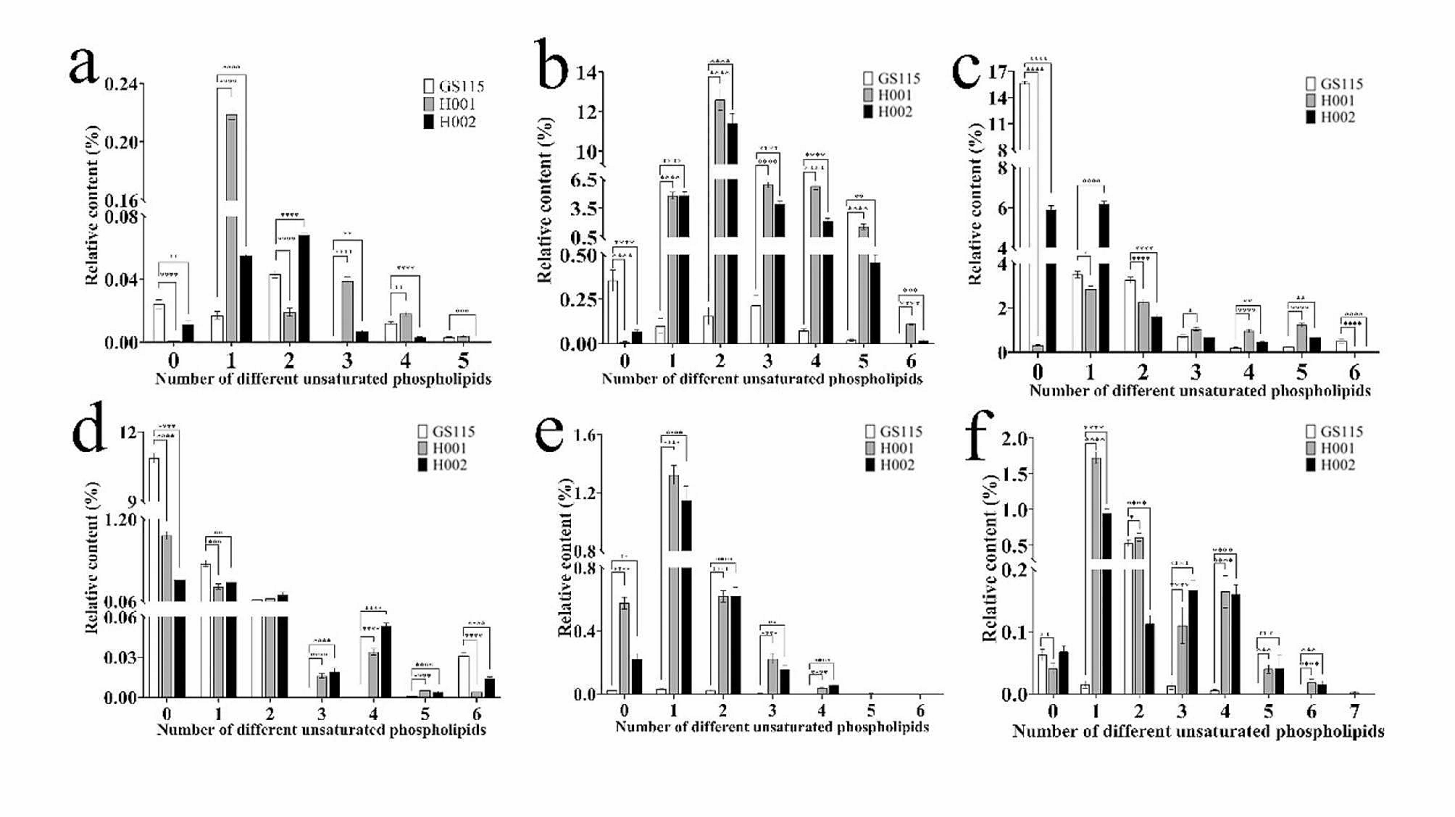
Number of different unsaturated phospholipids in *P. pastoris* GS115, H001 and H002. (**a**) phosphatidic acid (PA). (**b**) phosphatidylcholine (PC). (**c**) phosphatidylethanolamine (PE). (**d**) glycerol phosphatidic acid (PG). (**e**) phosphatidylinositol (PI). (**f**) phosphatidylserines (PS). Mean ± SEM are shown (*n* = 6). Error bars indicate standard deviation. * Represented *p* < 0.05, ** represented *p* < 0.01, *** represented *p* < 0.001 and **** represented *p* < 0.0001

Notably, the relative content of unsaturated glycerophospholipids in H001 and H002 increased significantly (Fig. 7a-f; Table 3). Most unsaturated glycerophospholipids, such as unsaturated PA, PC, PE, PG, PI, and PS, were notably elevated in H001 and H002 compared to GS115, likely associated with their improved performances.

**Table 3:** Phospholipids unsaturated index in *P. pastoris* GS115, H001 and H002

Lipid class	Phospholipids unsaturated index (%) ^a^		
	GS115	H001	H002
PA	0.17 ± 0.04	0.47 ± 0.06	0.23 ± 0.02
PC	1.44 ± 0.37	79.41 ± 5.12	50.42 ± 3.69
PE	17.11 ± 4.05	20.53 ± 3.30	21.90 ± 2.20
PG	0.94 ± 0.16	1.70 ± 0.19	1.00 ± 0.13
PI	0.09 ± 0.02	3.41 ± 0.62	3.41 ± 0.72
PS	0.59 ± 0.12	4.25 ± 0.46	2.61 ± 0.34

^a^$\text{U}\text{I}\left(\text{\%}\right)=\sum _{\text{i}=1}^{\text{n}}\left[\text{i}\cdot {\text{X}}^{\text{i}}\right];\text{i}=\text{1,2},\cdots ,\text{n};{\text{x}}^{\text{i}}$ for unsaturated double bond content

## Discussions

In yeast, *β*-D-glucans and mannan, the two major polysaccharides within the cell wall, made up approximately 85-90% of dry weight of cell wall, which comprised 26-32% of the total cell dry weight. Two types of *β*-D-glucans were discovered in yeast, including *β*-1,3-D-glucan (the major component accounting for 85%) and *β*-1,6-D-glucan. Firstly, *β*-1,3-glucan synthase was involved in the synthesis of *β*-1,3-glucan and its translocation out from the plasma membrane, and later, enzymes of the Gel/Gas/Phr protein family were responsible for *β*-1,3-glucan processing in the cell wall [51]. Mannan was biosynthesized by α-1,6-mannosyltransferase to form its main chain α-1,6-glycan and the side chain α-1,2-glycan was ligated by α-1,2-mannosyltransferase [52]. This study posited that *P. pastoris* GS115 synthesizes redundant cell wall polysaccharides, leading to excessive consumption of carbon sources. Consequently, reducing these polysaccharides could potentially result in more efficient carbon utilization, directing resources toward growth, protein expression, and biocatalysis. The primary objective of the current study was to validate this hypothesis and construct *P. pastoris* chassis hosts deficient in cell wall polysaccharides.

Both novel hosts in this study demonstrated accelerated growth and a higher carbon-to-biomass conversion ratio compared to GS115. The consequences could be associated with the redirection of the carbon source. Fewer carbon sources likely went towards the biosynthesis of cell wall polysaccharides, while more were directed toward cell growth and metabolism, a phenomenon supported by higher NADPH and ATP levels. These speculations require further verification in future studies.

Additionally, the mannan content in H001 and H002 showed a slight increase along with glucan decline (Fig. 1b). This suggests the existence of a compensation mechanism to maintain the balance between glucan and mannan in the cell wall of *P. pastoris*. This finding is in line with a previous report indicating that the composition and structure of the fungal cell wall can dynamically change due to the complexity of the cell wall biosynthesis process [53].

The sum of glucan and mannan contents in H001 decreased to 186.43 mg/g, and in H002, it further decreased to 176.87 mg/g, compared to GS115 (193.27 mg/g). This indicates a reduction in cell wall polysaccharides. There is also a correlation between cell wall polysaccharide content and cell size. The order for polysaccharide content is GS115 > H001 > H002, while for cell size, it is H002 > H001 > GS115. The changes of structural polysaccharides could influence cell wall rigidity [54]. Both H001 and H002 exhibited larger cell size, possibly resulting from the reduced cell wall rigidity. It is known that cell wall was widely involved in various yeast adaptation and tolerance to complex stresses. Especially, cell wall polysaccharides played critical roles in maintaining cell structure and conferring tolerance during fermentation cycle [55]. For an example in the highly ethanol-tolerant strain *K. marxianus* FIM1, its increased tolerance to ethanol was associated with transcription rewiring associated with cell wall synthesis, such as the upregulation of cell wall metabolism, notably *β*-1,6-glucan synthesis [56].

This work also found that the tolerance of H001 and H002 to methanol was not disrupted. When glycerol was exhausted in BMGY + BMMY culture, methanol was added immediately at 48 h (Fig. 1d). In the case of GS115 and H001, the biomass dropped slightly after methanol addition and rebounded 12 h later, indicating similar adaptation abilities. However, for H002, OD_600_ rebounded 8 h later, indicating a better tolerance to methanol than GS115.

Moreover, the detectable increase of unsaturated glycerophospholipids in H001 and H002 probably mitigated oxidative stress, a critical factor defining protein expression and catalytic reactions reported previously [57].

Both H001 and H002 exhibited higher growth rates and attained higher peak biomass compared to GS115. The increased carbon source consumption in H001 and H002 was primarily attributed to their enhanced growth, while their specific carbon source consumption was not higher than that of GS115. Elevating the conversion rate of glycerol, glucose, and methanol to biomass, protein, and biochemicals is crucial for achieving high expression efficiency and improved cost-effectiveness in large-scale *P. pastoris* fermentation. The findings of this study suggest that reducing cell wall polysaccharides is an effective strategy for enhancing carbon source utilization efficiency. In comparison to the original GS115, the novel chassis H001 and H002 exhibited not only enhanced performance in terms of carbon utilization and growth rate but also improved biomanufacturing efficiency, as evidenced by increased production of *hegf*, SAM, and ERG.

Numerous studies have focused on the expression of *hegf*, primarily reported in *E. coli*. In Eissazadeh’s study, H002/pGAPZα A-*hegf* produced the highest *hegf* content at 40.37 µg/mL, a substantial improvement over the previous *hegf* level of 2.27 µg/mL in *P. pastoris* GS115 [58]. In this study, ERG was synthesized for the first time in *P. pastoris*. Notably, H001 exhibited the highest ERG yield, amounting to 75.85 mg/L, comparable to levels observed in *S. cerevisiae* [44].

Recently, sustainability moved high up worldwide on the political agenda [59]. There were huge consumption of glycerol, glucose and methanol in large-scale fermentation of *P. pastoris*. The use of glycerol and glucose will clash with the food needs of a population still increasing in the face of drought, soil destruction, and climate change more generally, and the use of methanol will be limited by the gradual shortage of fossil fuels. Therefore, it was of great significance to elevate their conversion rate to biomass, protein and biochemicals.

The study findings suggested that the improved performance of the two chassis hosts resulted from increased NADPH and ATP availability, along with reduced oxidative damage attributed to higher concentrations of unsaturated lipids. The deficiency in glucan in H001 and H002 leading to changes in lipid composition, unsaturation degree, and relative content prompts further investigation. It reflects an unclear interplay between lipid and glucan synthesis, as they share common precursor molecules. An alternative hypothesis suggests that the reduction in glucan content, considered as cell wall damage, might be sensed by *P. pastoris*. In response, the organism induces cellular mechanisms, possibly involving the regulation of membrane lipid biosynthesis, to mitigate oxidative damage. It was known that cell wall damage can activate MAPK signaling pathways, which made a response via activating the repair of cell wall [54]. Next, the study tends to identify the pathways responsible for transmitting stress signals resulting from the damaged cell wall in H001 and H002. Additionally, knowledge on stress responses, including changes in gene expression and transcripts will be clarify to further understand the cellular mechanisms involved in future plan.

## Conclusion

This study successfully developed innovative chassis hosts. Two engineered *P. pastoris* chassis cells (H001 and H002) deficient in glucan biosynthesis exhibited enhanced carbon source utilization, accelerated growth rates, and improved bioproduction efficiency compared to the original *P. pastoris* GS115. This superior performance included elevated expression levels of GFP and *hegf*, along with increased biosynthesis yields of SAM and ERG. Further investigation unveiled potential contributors to the improved performance, including higher levels of NADPH and ATP, as well as reduced oxidative damage due to increased unsaturated lipids in both chassis hosts. These modified chassis hosts demonstrate significant potential for advancing sustainable biological manufacturing in the future.

### Electronic supplementary material

Below is the link to the electronic supplementary material.

Supplementary Material 1

Supplementary Material 2

Supplementary Material 3

Supplementary Material 4

Supplementary Material 5

Supplementary Material 6

Supplementary Material 7
