# Supplementary material for: Impact of cell wall polysaccharide modifications on the performance of Pichia pastoris: novel mutants with enhanced fitness and functionality for bioproduction applications
Source: Microb Cell Fact. 2024 Feb 17;23:55. doi: 10.1186/s12934-024-02333-0 (PMC10874062; doi:10.1186/s12934-024-02333-0)

Fig. S1 Construction schematic of the plasmids under control of P_AOX_ (right) and P_GAP_ (left) respectively: (a) pGAPZ A-*gfp* and pPICZ A-*gfp*; (b) pGAPZα A-*hegf* and pPICZα A-*hegf*; (c) pGAPZ A-*sam2* and pPICZ A-*sam2*; (d) pGAPZ A-*egt12* and pPICZ A-*egt1E*; (e) pGAPZ A-*egt1E* and pPICZ A-*egt12*.


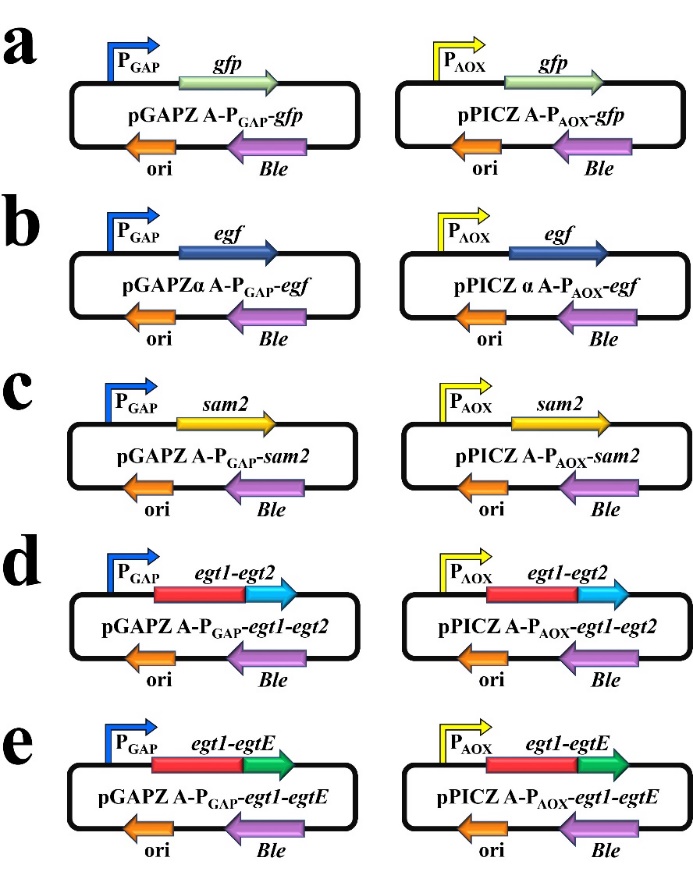

Supplement: Supplementary file 1 — Supplementary Material 1 [file 12934_2024_2333_MOESM1_ESM.docx]
