# Supplementary material for: Impact of cell wall polysaccharide modifications on the performance of Pichia pastoris: novel mutants with enhanced fitness and functionality for bioproduction applications
Source: Microb Cell Fact. 2024 Feb 17;23:55. doi: 10.1186/s12934-024-02333-0 (PMC10874062; doi:10.1186/s12934-024-02333-0)

Fig. S2 The content of malondialdehyde (MDA) during cultivation of *P. pastoris* GS115, H001 and H002 controlled by P_GAP_ (a) and P_AOX_ (b) respectively. Mean ± SEM are shown (n = 6). Error bars indicate standard deviation. ** Represented p < 0.01, *** represented p < 0.001 and **** represented p < 0.0001.


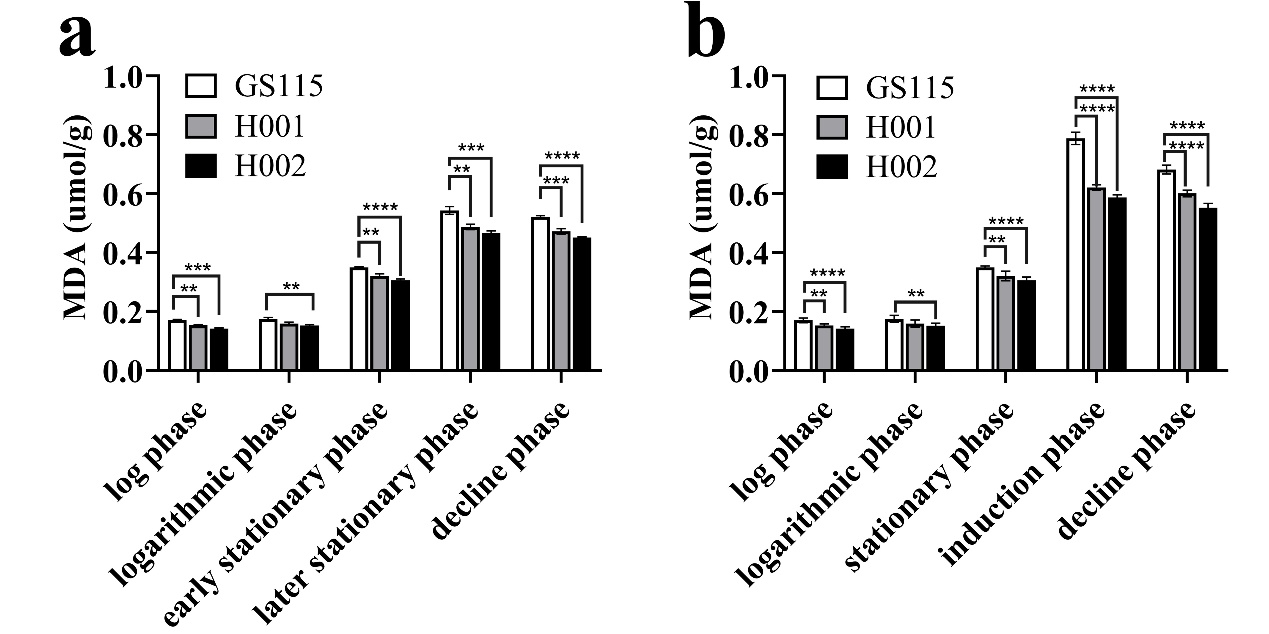

Supplement: Supplementary file 2 — Supplementary Material 2 [file 12934_2024_2333_MOESM2_ESM.docx]
