# Supplementary material for: Impact of cell wall polysaccharide modifications on the performance of Pichia pastoris: novel mutants with enhanced fitness and functionality for bioproduction applications
Source: Microb Cell Fact. 2024 Feb 17;23:55. doi: 10.1186/s12934-024-02333-0 (PMC10874062; doi:10.1186/s12934-024-02333-0)

Fig. S3 Numbers of different classes of lipids in GS115, H001 and H002. phosphatidic acid (PA); phosphatidylcholine (PC); phophatidylethanolamine (PE); glycerol phosphatidic acid (PG); phosphatidylinositol (PI); phosphatidylserines (PS); diacylglycerol (DG); triacylglycerol (TG); ceramides (Cer); sphingosine (So).


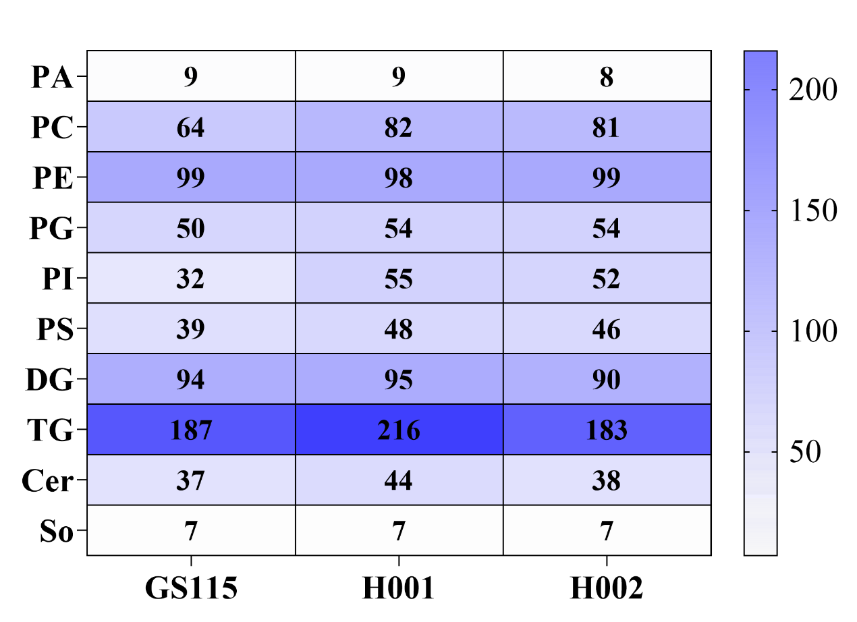

Supplement: Supplementary file 3 — Supplementary Material 3 [file 12934_2024_2333_MOESM3_ESM.docx]
