# Supplementary material for: Impact of cell wall polysaccharide modifications on the performance of Pichia pastoris: novel mutants with enhanced fitness and functionality for bioproduction applications
Source: Microb Cell Fact. 2024 Feb 17;23:55. doi: 10.1186/s12934-024-02333-0 (PMC10874062; doi:10.1186/s12934-024-02333-0)

Fig. S4 The relative intensity of different classes of lipids in GS115, H001 and H002. (a) phosphatidylcholine (PC); phosphatidylethanolamine (PE); glycerol phosphatidic acid (PG); phosphatidylinositol (PI); phosphatidylserines (PS); phosphatidic acid (PA). (b) diacylglycerol (DG); triacylglycerol (TG). (c) ceramides (Cer); sphingosine (So). Mean ± SEM are shown (n = 6). Error bars indicate standard deviation. *** Represented p < 0.001 and **** represented p < 0.0001.


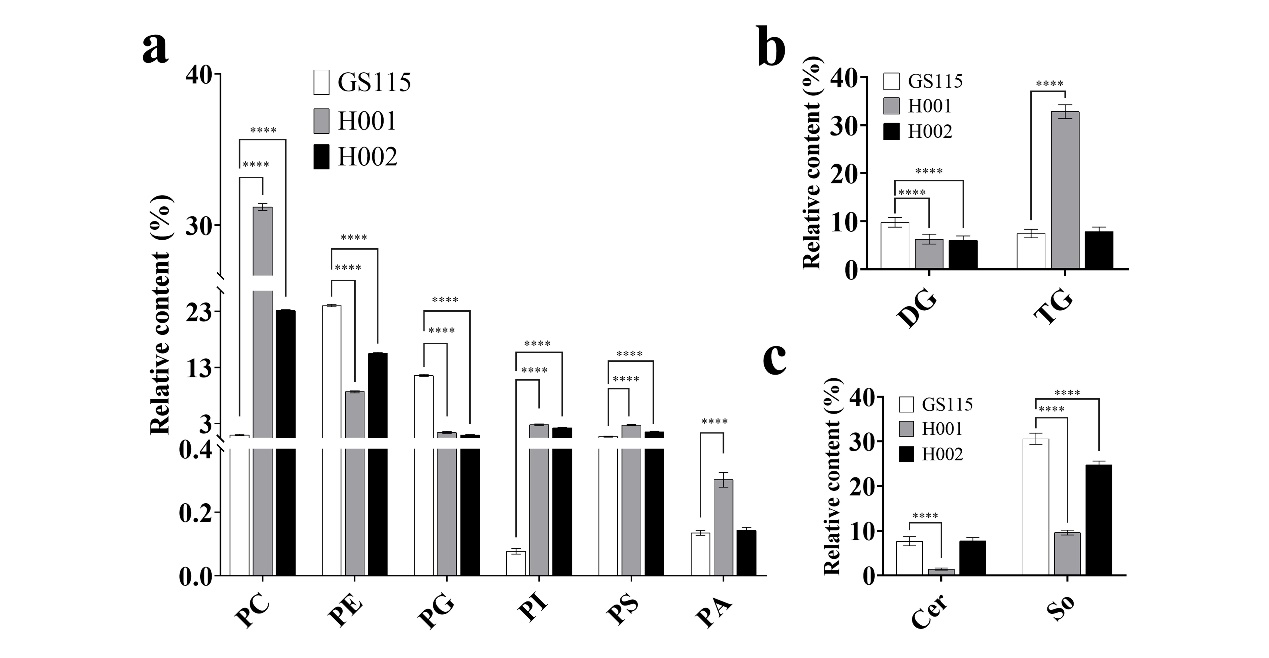

Supplement: Supplementary file 4 — Supplementary Material 4 [file 12934_2024_2333_MOESM4_ESM.docx]
