# Supplementary material for: Impact of cell wall polysaccharide modifications on the performance of Pichia pastoris: novel mutants with enhanced fitness and functionality for bioproduction applications
Source: Microb Cell Fact. 2024 Feb 17;23:55. doi: 10.1186/s12934-024-02333-0 (PMC10874062; doi:10.1186/s12934-024-02333-0)
Supplement: Supplementary file 5 — Supplementary Material 5 [file 12934_2024_2333_MOESM5_ESM.docx]

Table S1 The plasmids used in the present study.

| **Plasmids** | **Descriptions** |
| --- | --- |
| pPpT4-pHTX1-hsCas9 | Knock-out plasmid with pHTX1, Cas9-Homo sapiens and Zeocin |
| pPpT4-pHTX1-hsCas9- *PAS_chr1-3_0225* | harboring sgRNA of *PAS_chr1-3_0225* |
| pPpT4-pHTX1-hsCas9- *PAS_chr2-1_0661* | harboring sgRNA of *PAS_chr2-1_0661* |
| pGAPZ A | Plasmid carrying P_GAP_, and Zeocin |
| pPICZ A | Plasmid carrying P_AOX_, and Zeocin |
| pGAPZα A | Plasmid carrying P_GAP_, α factor and Zeocin |
| pPICZα A | Plasmid carrying P_AOX_, α factor and Zeocin |
| pGAPZ A-*gfp* | pGAPZ A harboring *gfp* under P_GAP_ |
| pPICZA-*gfp* | pPICZ A harboring *gfp* under P_AOX_ |
| pGAPZα A-*hegf* | pGAPZα A harboring *hegf* under P_GAP_ |
| pPICZα A-*hegf* | pPICZα A harboring *hegf* under P_AOX_ |
| pGAPZ A-*sam2* | pGAPZ A harboring *sam2* under P_GAP_ |
| pPICZA-*sam2* | pPICZ A harboring *sam2* under P_AOX_ |
| pGAPZ A-*egt12* | pGAPZ A harboring *egt12* under P_GAP_ |
| pGAPZ A-*egt1E* | pGAPZ A harboring *egt1E* under P_GAP_ |
| pPICZ A-*egt12* | pPICZ A harboring *egt12* under P_AOX_ |
| pPICZ A-*egt1E* | pPICZ A harboring *egt1E* under P_AOX_ |
