# Supplementary material for: Impact of cell wall polysaccharide modifications on the performance of Pichia pastoris: novel mutants with enhanced fitness and functionality for bioproduction applications
Source: Microb Cell Fact. 2024 Feb 17;23:55. doi: 10.1186/s12934-024-02333-0 (PMC10874062; doi:10.1186/s12934-024-02333-0)
Supplement: Supplementary file 6 — Supplementary Material 6 [file 12934_2024_2333_MOESM6_ESM.docx]

Table S2 The primers used in the present study.

| **Primers** | **Primer sequence** |
| --- | --- |
| *PAS_chr1-3_0225*-up-F | CTGCCGCCTCTTTCAAGCAC |
| *PAS_chr1-3_0225*-up-R | ATAGATCAATCTATCGATCTAGAGAGCTAGCTAGG GAAAGGATTGCAGGGCGTAGGAGGTAT |
| *PAS_chr1-3_0225*-down-F | AGCTAGCTCTCTAGATCGATAGATTGATCTATGGAA CCGTTCACAGGGAGAAATCAAATGAT |
| *PAS_chr1-3_0225*-down-R | GCTGGATGCCCCTGGAGTAGTTGATGACT |
| *PAS_chr1-3_0225*-sgRNA-F | CAATGAGAGTCAAGAAATCTGTTTTAGAGCTAGAA ATAGCAAGTTAAAATAA |
| *PAS_chr1-3_0225*-sgRNA-R | AGATTTCTTGACTCTCATTGGACGAGCTTACTCGTTT CGTCC |
| *PAS_chr2-1_0661*-up-F | GAACAACCGGAACAGAATGTGGTTC |
| *PAS_chr2-1_0661*-up-R | ATAGATCAATCTATCGATCTAGAGAGCTAGCTTCAAACAAACGGAGCAAATCGGC |
| *PAS_chr2-1_0661*-down-F | AGCTAGCTCTCTAGATCGATAGATTGATCTATTTGGGACATTCAATCAATGGTGGAAAAGAC |
| *PAS_chr2-1_0661*-down-R | CTGCCGCCTCTTTCAAGCAC |
| *PAS_chr2-1_0661*-sgRNA-F | ATGGATGATAGCCCTCAGAAGTTTTAGAGCTAGAAATAGCAAG |
| *PAS_chr2-1_0661*-sgRNA-R | TTCTGAGGGCTATCATCCATGACGAGCTTACTCGTTTCGTC |
| pGAPZ A-*gfp*-F | GAACAACTATTTCGAAACGATGAGTAAAGGAGAAGAACTTTTCACTGG |
| pGAPZ A-*gfp*-R | GGCTGGGCCACGTGAATTCCTATTTGTATAGTTCATCCATGCC |
| pPICZ A-*gfp*-F | GTTTCGAATAATTAGTTGTTTTTTGATC |
| pPICZ A-*gfp*-R | AATAGGCCGCCAGCTTTCTAGAAC |
| pGAPZα A-*hegf*-F | GTTTCGAAATAGTTGTTCAATTGATTG |
| pGAPZα A-*hegf*-R | GATCAAAAAACAACTAATTATTCGAAACGATGAGTAAAGGAGAAGAACTTTTCACTG |
| pPICZα A-*hegf*-F | GTTCTAGAAAGCTGGCGGCCTATTTGTATAGTTCATCCATGCCATGTGATAT |
| pPICZα A-*hegf*-R | GAACAACTATTTCGAAACGATGAGTAAAGGAGAAGAACTTTTCACTGG |
| pGAPZ A-*sam2*-F | GGCTGGGCCACGTGAATTCCTATTTGTATAGTTCATCCATGCC |
| pGAPZ A-*sam2*-R | GTTTCGAATAATTAGTTGTTTTTTGATC |
| pPICZ A-*sam2-*F | AATAGGCCGCCAGCTTTCTAGAAC |
| pPICZ A*-sam2-*R | GCCGCCAGCTTTCTAGAACA |
| pGAPZ A*-egt1-*F | TGAACAACTATTTCGAAACGATGCCATCTGCTGAATCTATGACTC |
| pGAPZ A*-egt1-*R | CAATTCAACAGTAGTAGCAACCATTTACAAATCTCTAACAACTCTAG |
| pGAPZ A*-egt2-*F | CTAGAGTTGTTAGAGATTTGTAAATGGTTGCTACTACTGTTGAATTG |
| pGAPZ A*-egt2-*R | CGGCTGGGCCACGTGAATTCTTAAGCAGATTCTTTATATTCACCTTTAGC |
| pGAPZ A*-egtE-*F | GAGTTGTTAGAGATTTGTAAGTGATGCTGGCGCAGCAG |
| pGAPZ A*-egtE-*R | CGGCTGGGCCACGTGAATTCTTACGGCGCTTCGCGCAG |
| pPICZ A-*egt1-*F | GATCTGAATAGCGCCGTCGACATGCCATCTGCTGAATCTATGACTC |
| pPICZ A-*egt1-R* | CAATTCAACAGTAGTAGCAACCATTTACAAATCTCTAACAACTCTAG |
| pPICZ A-*egt2-*F | CTAGAGTTGTTAGAGATTTGTAAATGGTTGCTACTACTGTTGAATTG |
| pPICZ A-*egt2-R* | ATGATGATGATGATGGTCGACTTAAGCAGATTCTTTATATTCACCTTTAGC |
| pPICZ A-*egtE-*F | GAGTTGTTAGAGATTTGTAAGTGATGCTGGCGCAGCAG |
| pPICZ A-*egtE-R* | TGATGATGATGATGGTCGACTTACGGCGCTTCGCGCAG |
