# Supplementary material for: Impact of cell wall polysaccharide modifications on the performance of Pichia pastoris: novel mutants with enhanced fitness and functionality for bioproduction applications
Source: Microb Cell Fact. 2024 Feb 17;23:55. doi: 10.1186/s12934-024-02333-0 (PMC10874062; doi:10.1186/s12934-024-02333-0)
Supplement: Supplementary file 7 — Supplementary Material 7 [file 12934_2024_2333_MOESM7_ESM.docx]

Table S3 The number of main lipids variation in H001 and H002 in comparison to GS115.

| Lipid category | The mumber of changed lipid (s) | | |
| --- | --- | --- | --- |
|  | GS115 | H001 | H002 |
| Glycerophospholipid | 0 | +53 | +47 |
| Glyceride | 0 | +30 | -8 |
| Sphingolipid | 0 | +7 | +1 |
